# Integrated Single‐Cell and Bulk Transcriptomic Analysis Reveals an Endothelial Gene Signature Shaping EndMT and Prognosis in Hepatocellular Carcinoma

**DOI:** 10.1155/humu/4041113

**Published:** 2026-06-17

**Authors:** Fuqun Wei, Xiang You, Zhisheng Chen, Yiping Chen, Zhongwu Chen

**Affiliations:** ^1^ Department of Interventional Radiology, the First Affiliated Hospital, Fujian Medical University, Fuzhou, China, fjmu.edu.cn; ^2^ Department of Interventional Radiology, National Regional Medical Center, Binhai Campus of the First Affiliated Hospital, Fujian Medical University, Fuzhou, China, fjmu.edu.cn

**Keywords:** endothelial cells, endothelial-to-mesenchymal transition, hepatocellular carcinoma, immune response, prognosis

## Abstract

**Background:**

Hepatocellular carcinoma (HCC) is one of the most prevalent malignant tumors of the liver, with increasing incidence and mortality rates globally. This study investigates the role of endothelial cells (ECs) in the progression of HCC, particularly focusing on the process of endothelial‐to‐mesenchymal transition (EndMT) and its underlying molecular mechanisms.

**Methods:**

We utilized single‐cell RNA sequencing (scRNA‐seq) and multiomics analyses to explore the involvement of ECs in HCC and their transformation mechanisms. The study emphasized the potential application of EC‐related genes (ECRGs) in prognostic evaluation for HCC patients. Furthermore, HCC organoids were utilized to validate the functional relevance of selected ECRGs.

**Results:**

ECs play a critical role in the progression of HCC, particularly through mechanisms related to EndMT and its influence on patient prognosis. We identified 19 key ECRGs that are pivotal to the initiation and development of HCC and constructed a robust prognostic model using LASSO regression analysis. Notably, this gene signature effectively stratifies HCC subtypes and reveals significant differences in immune cell infiltration and immune checkpoint gene expression among EC‐related prognostic groups, underscoring its potential relevance in guiding immunotherapy, sorafenib therapy, and transarterial chemoembolization (TACE) outcomes. Furthermore, the coculture system of HCC and vascular organoids successfully mimicked the EndMT process, revealing spatial colocalization of three ECRGs—MPZL2, KITLG, and PCDH1—with established EndMT markers, suggesting their potential as therapeutic targets in tumor‐associated endothelial plasticity.

**Conclusions:**

This study emphasizes ECs’ role in HCC progression and how EndMT affects prognosis. HCC organoids revealed ECRGs linked to EndMT, like MPZL2, KITLG, and PCDH1, as potential therapeutic targets.

## 1. Introduction

In HCC, ECs are involved in tumor angiogenesis, vascular remodeling, and immune cell trafficking. Abnormal activation of tumor‐associated ECs contributes to the formation of disorganized vasculature, which supports tumor growth and metastasis [1]. Furthermore, ECs regulate the infiltration and polarization of immune cells by expressing chemokines, adhesion molecules, and immune checkpoint ligands, thereby playing an immunomodulatory role in the tumor microenvironment (TME) ([2–4]; [5]). Recent advances in scRNA‐seq have revealed the remarkable heterogeneity of ECs within liver tumors, offering new insights into their functional diversity and plasticity ([6–8]. [9]).

A key manifestation of this plasticity is the process of EndMT, through which ECs lose their endothelial phenotype and acquire mesenchymal characteristics. EndMT represents a critical shift in cell identity, characterized by downregulation of endothelial markers, such as CD31 and VE‐cadherin, and upregulation of mesenchymal markers including vimentin, *α*‐SMA, and fibronectin. This phenotypic switch is driven by multiple tumor‐associated stimuli, such as TGF‐*β*, hypoxia, and inflammatory cytokines, and has been shown to contribute to several malignant processes in HCC ([10]. [11]). Through EndMT, ECs can enhance extracellular matrix deposition, increase tumor stiffness, and support vascular mimicry, all of which facilitate tumor progression, immune evasion, and therapeutic resistance. Moreover, mesenchymal‐like ECs may create a fibrotic and immunosuppressive microenvironment that hinders immune cell infiltration and limits the efficacy of immunotherapies [12].

Given the central role of ECs and the EndMT process in shaping the TME and influencing HCC outcomes, a deeper understanding of their molecular characteristics is urgently needed. In this study, we utilized scRNA‐seq data to identify endothelial cell–specific marker genes in HCC, revealing potential signatures reflective of EC heterogeneity. To further investigate EndMT‐related gene expression patterns, we conducted weighted gene coexpression network analysis (WGCNA) on bulk RNA‐seq data from The Cancer Genome Atlas (TCGA) to identify gene modules associated with EndMT activity. Subsequently, we integrated EC signatures and EndMT‐related genes to develop a prognostic gene model consisting of nine related molecules. Additionally, we explored its associations with overall survival, immune cell infiltration, and drug sensitivity. Our findings provide new insights into the interplay between endothelial plasticity and the immunosuppressive microenvironment in HCC, offering potential biomarkers and therapeutic targets for improving clinical outcomes in liver cancer patients.

## 2. Materials and Methods

### 2.1. Data Collection

scRNA‐seq data GSE149614 from 10 HCC patients were retrieved from GEO (https://www.ncbi.nlm. http://nih.gov/geo/), including nontumor liver (NTL), primary tumor (PT), portal vein tumor thrombus (PVTT), and metastatic lymph node (MLN) tissues. Nine HCC RNA‐seq datasets were collected, with a training cohort of 373 samples from TCGA‐LIHC (https://portal.gdc.cancer.gov/repository) and validation cohorts of 231 cases from the JP Project of the International Cancer Genome Consortium (ICGC‐LIRI‐JP) and 221 samples from GSE14520. Six GEO datasets (GSE109211, GSE104580, GSE279750, GSE202069, GSE181946, and GSE215011) evaluated the EC signature’s therapeutic efficacy, including 67 cases treated with sorafenib and 147 with TACE, while 51 cases treated with PD‐1/PD‐L1 inhibitors were analyzed after batch effect removal.

### 2.2. Single‐Cell Data Processing and Annotation

We used the “Create Seurat Object” function from Seurat (Version 5.0.1) [13] to import sequencing data, setting parameters for gene expression and filtering cells based on RNA count (≥ 700), feature number (≥ 500), and mitochondrial gene ratio (< 20%). After normalizing the data with “NormalizeData,” we applied “RunTSNE” for dimensionality reduction with a clustering resolution of 0.3, visualizing 24 clusters and identifying 6 cell types via SingleR (Version 2.4.1) [14] and manual annotation.

### 2.3. CopyKAT Analysis

To further investigate the characteristics and functions of malignant EC subpopulations, we analyzed single‐cell data using the R package CopyKAT (Version 1.1.0) [15] to assess the proportion of malignant (aneuploid) cells across different groups and extracted malignant endothelial cells for subsequent analyses.

### 2.4. Scoring EC Features Across Tissue Origins

The proliferation scores of ECs in different groups were calculated based on the expression levels of signature genes associated with tumor proliferation ability (MKI67, IGF1, ITGB2, PDGFC, JAG1, and PHGDH), as reported in previous studies [16, 17]. Meanwhile, the migration scores were determined by quantifying the expression of signature genes indicative of tumor migration ability (VIM, SNAI1, MMP9, AREG, ARID5B, and FAT1), as described in previous studies ([18–20]). Additionally, based on previous studies [21–23], we examined the expression levels of cancer stem cell (CSC) marker CD44, endothelial cell markers (CDH15, PECAM1, TEK, and VWF), and mesenchymal cell markers (ACTA2, TAGLN, VIM, and S100A4). In line with prior literature [24], both functional scores were defined as the average normalized expression of the corresponding genes.

### 2.5. Immune Cell Infiltration and Survival Analysis

The abundance of immune and nonimmune stromal cell populations was estimated from gene expression data using the MCPcounter R package [25]. Tumor samples from TCGA‐LIHC, GSE14520, and ICGC‐LIRI‐JP were categorized into high and low groups based on endothelial cell abundance median, followed by survival analysis with the Kaplan–Meier formula in R’s “Survival” package and visualization using “survminer.”

### 2.6. Identification and Validation of EC Prognostic Gene Signature

We used “FindAllMarkers” to identify DEGs (|log*F* *C*| > 1 and adjusted *p* value < 0.05) of ECs in PT, PVTT, and MLN tissues compared with ECs in NTL tissue, labeling these DEGs as the EC‐related gene set. Using the IOBR package (Version 2.2.0) ([26]), we applied single‐sample gene set enrichment analysis (ssGSEA) to score TCGA‐LIHC tumor samples for the EndMT gene set from GeneCards (https://www.genecards.org/), including only genes with scores over 2. Samples were categorized into high‐ and low‐scoring groups based on median scores. We conducted WGCNA with the WGCNA R package (Version 1.74) [27], calculating gene correlation coefficients and ensuring scale‐free network adherence. A hierarchical clustering tree was built, with branches representing distinct gene modules assigned unique colors. We assessed module significance, filtering the top 50% of genes by variance, setting a minimum of 60 genes per module, an optimal soft threshold of 6, a scale‐free fitting index of 0.9, and a merging cut height of 0.2. We analyzed correlations between endothelial cell scores and modules, identifying module‐characteristic genes. Modules with *p* values under 0.05 were designated as module genes, forming the EndMT‐related gene set. We used the R package survival for univariate Cox regression to evaluate gene influence on prognosis using TCGA‐LIHC data, aiming to identify independent prognostic factors with *p* < 0.05 and intersected prognostic genes with endothelial and EndMT gene sets to find endothelial genes related to HCC. Following that, we conducted LASSO regression on intersection genes using glmnet (Version 4.1.8) [28] with 10 cycles, visualizing outcomes through a risk model and variable trajectory diagrams. A stepwise multivariate Cox model identified key survival‐related genes for a prediction model based on B cell markers. The risk score was calculated using the expression and regression coefficients of these genes, dividing samples into high‐ and low‐risk groups based on the median score. Validation was performed using three GEO datasets and the ICGC‐LIRI‐JP database, with the Kaplan–Meier analysis assessing overall survival differences and ROC curves evaluating model performance at 1, 3, and 5 years.

### 2.7. Consensus Clustering

Consensus clustering method using R package ConsensusClusterPlus [29] was used to identify different disease subtypes of LIHC samples based on prognostic genes from TCGA‐LIHC. In this process, the number of clusters was set between 2 and 9, and 80% of the total samples were repeated 50 times, clusterAlg = “km,” distance = “euclidean.”

### 2.8. Correlation Analysis of EndMT Genes

EndMT‐signature genes (MMP9, ACTA2, ZEB2, PECAM1, MMP2, COL3A1, COL1A1, VIM, SNAI1, ZEB1, KDR, FLT1, SNAI2, FN1, and CDH2) were derived from previous literature reports [12, 21, 23]. The significant correlations between subtypes and EndMT‐related genes were visualized using the corrplot function from the corrplot package in R.

### 2.9. Tumor Immune Dysfunction and Exclusion (TIDE), Immune Checkpoint, and Immune Infiltration Analysis

The TIDE immunoscore for HCC samples was obtained by analyzing the dataset on the TIDE website (http://tide.dfci.harvard.edu) [30, 31], predicting tumor treatment responses. Immunologic checkpoint genes (ICGs) were retrieved from the literature [32], with names listed in Table S1. The Mann–Whitney *U* test analyzed ICG expression differences between clusters, generating a comparison plot. Immune cell infiltration was assessed using six TME analysis algorithms integrated in the IOBR R package (Version 3.6.0).

### 2.10. Enrichment Analysis

Gene Set Enrichment Analysis (GSEA) was performed using clusterProfiler [33] to probe KEGG [34] and GO [35] gene set libraries. GSEA was performed using the list with GSEA software [36].

### 2.11. Drug Sensitivity Analysis

We used the pRRophetic algorithm [37] to predict the sensitivity of two groups of LIHC patients to common anticancer drugs or small molecule compounds by calculating the IC50 value based on the LIHC samples, and the results were shown by a group comparison diagram.

### 2.12. Establishment of a Vascularized HCC Organoid Model and Induction of EndMT

This study was conducted in accordance with the ethical guidelines of the 1975 Declaration of Helsinki and was approved by the Ethics Committee of the Hospital (No. MRCTA, ECFAH of FMU [2024] 469). Tumor tissues were obtained from four HCC patients who underwent surgical resection. Tumor tissues were collected and placed in a preservation solution (AIMINGMED, MasterAim #100‐049) at 4°C and processed within 24 h to maintain cell viability. Necrotic and nontumor components were removed, and tissues were minced in a medium with antibiotics. Samples were digested at 37°C for 15–45 min and monitored microscopically, and digestion was stopped with FBS when single cells appeared. The suspension was filtered, centrifuged, and washed; if red blood cells were present, lysis was performed. The cell pellet was resuspended in Matrigel (gel content > 70*%*), seeded into plates, gelled at 37°C, and cultured in liver cancer organoid medium (AIMINGMED, 10‐100‐398), with imaging every 2 days and medium refreshed every 3 days. Human pluripotent stem cells (hPSCs) were cultured in mTeSR1 medium and passaged at a 1:6 ratio upon 70%–80% confluence. EBs were formed in low‐attachment dishes for 2 days, followed by mesoderm differentiation with BMP4 (30 ng/mL), activin A (50 ng/mL), and bFGF (5 ng/mL) for 2 days and endothelial induction with VEGF‐A (50 ng/mL) and forskolin (5 *μ*M) for 4 days. Cells were then aggregated in collagen and Matrigel for 3D vascular network assembly, maintained for 14–21 days with medium changes every 2–3 days. For tumor angiogenesis modeling, liver cancer organoids were cocultured with vascular organoids in Matrigel‐based 3D scaffolds. Organoids were mixed at a 1:1–1:3 ratio depending on the experimental objective. Direct physical contact was ensured by embedding both types of organoids in the same matrix. The culture medium was supplemented with angiogenic factors (e.g., VEGF and FGF) and TME‐mimicking cytokines. TGF‐*β* was added at a concentration of 5 ng/mL for 5 days to induce EndMT. EndMT induction was validated via real‐time quantitative PCR (RT‐qPCR) using an ABI 7500 system with 40 cycles (95°C for 10 s, 58°C for 15 s, and 72°C for 35 s). Primer sequences are listed in Table S2. Statistical analysis was performed using a paired Student’s *t*‐test, and *p* values were reported accordingly.

### 2.13. IF Staining

Paraffin‐embedded sections were deparaffinized, rehydrated, and subjected to heat‐induced epitope retrieval using Tris‐EDTA buffer (pH 9.0). Endogenous peroxidase activity was blocked with hydrogen peroxide. Sections were then blocked with 5% BSA and incubated with primary antibodies overnight at 4°C, followed by secondary antibody incubation at 37°C for 30 min. Tyramide signal amplification was performed using TSA‐488 and TSA‐Cy3 reagents. Nuclei were counterstained with DAPI. All images were acquired on a Leica SP8 confocal microscope and analyzed using ImageJ software. The antibodies used are listed in Table S3.

## 3. Results

### 3.1. Tumor‐Infiltrating ECs Are Associated With a Favorable Prognosis and Contribute to HCC Progression Through EndMT

To confirm the prognostic significance of ECs in HCC, we first employed the MCPcounter algorithm to estimate the abundance of tumor‐infiltrating ECs in bulk RNA‐seq samples. Subsequently, we classified the samples from TCGA‐LIHC, GSE14520, and ICGC‐LIRI‐JP into high‐endothelial and low‐endothelial groups. The Kaplan–Meier analysis demonstrated a significant disparity between these two groups (*p* < 0.05) (Figure 1a). To further elucidate the role of ECs in the initiation and progression of HCC, we conducted an in‐depth analysis of single‐cell sequencing data from GSE149614. We applied t‐SNE for dimensionality reduction and visualization. At a resolution of 0.3, we obtained 24 independent clusters. Using the R package SingleR combined with manual annotation, we identified the cell clusters as a total of six cell types (Figure 1b), namely, hepatocytes, myeloid cells, ECs, B cells, fibroblasts, and T/NK cells. The expression patterns of marker genes across six cell subpopulations were visualized by a dot plot (Figure 1c). We found that the proportion of ECs in PVTT and MLN exhibited a lower compared to that in NTL and PT (Figure 1d). By using CopyKAT to identify and extract malignant ECs (Figure 1e), we observed a marked increase in the proportion of malignant ECs in both PT and PVTT (Figure 1f). We analyzed the proliferation, migration ability, and stemness of malignant ECs in different groups. It was revealed that the proliferation, migration, and stemness of EC in PT, PVTT, and MLN were enhanced compared to those of EC in NTL (Figure 1g–i). EndMT is one of the crucial mechanisms by which ECs promote the metastasis of HCC. While endothelial markers (CDH5, PECAM1, TEK, and VWF) showed no significant intergroup differences in expression (Figure 1j), ECs in the PT group exhibited significantly upregulated expression of mesenchymal markers (ACTA2, TAGLN, and S100A4) compared to all other groups (Figure 1k). These findings provide evidence that ECs may drive HCC progression via EndMT.

Figure 1(a) Kaplan–Meier analysis demonstrated a significant disparity between high‐endothelial abundance and low‐endothelial abundance groups in TCGA‐LIHC, GSE14520, and ICGC‐LIRI‐JP cohort. (b) t‐SNE plot showing 24 clusters in the GSE149614 dataset (left) and corresponding classification into six identified cell types (right). (c) Dot plot of cell cluster marker gene expression. (d) Proportions of annotated cell types across different tissue sources. (e, f) tSNE plot showing CopyKAT‐identified aneuploid (malignant) cells and their proportions across different tissue sources. (g–i) Violin plots showing the migratory, invasive capacity, and stemness of malignant ECs across different tissue sources. (j) Violin plots comparing the expression of endothelial markers (CDH5, PECAM1, TEK, and VWF) across different tissue sources. (k) Violin plots comparing the expression of mesenchymal markers (ACTA2, TAGLN, VIM, and S100A4) across different tissue sources. ns: *p* > 0.05,  ^∗^ *p* < 0.05,  ^∗∗^ *p* < 0.01,  ^∗∗∗^ *p* < 0.001.

**Figure:**
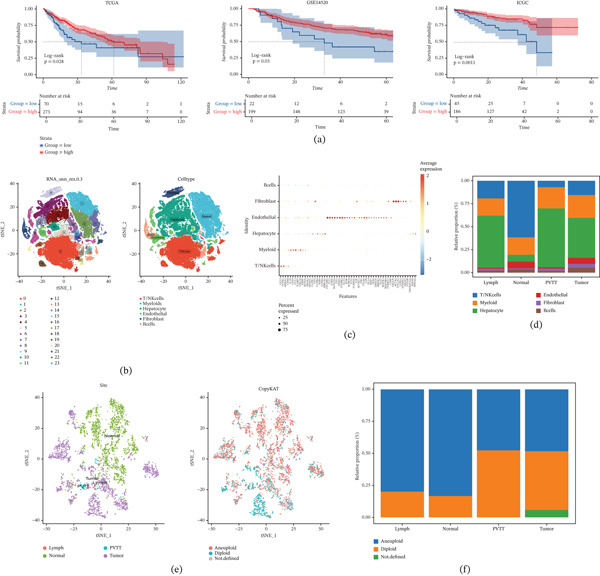
(A)

**Figure:**
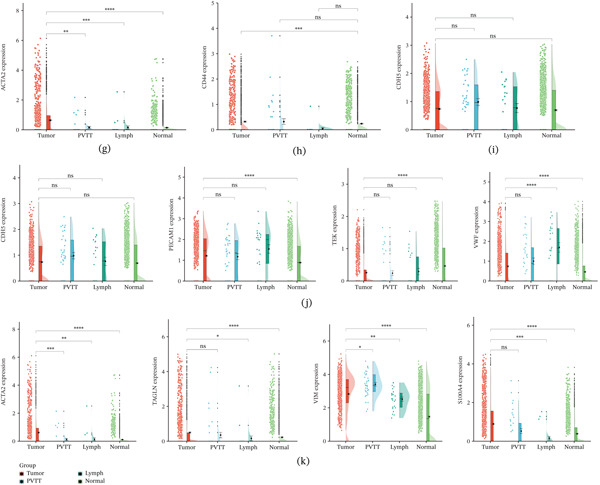
(B)

### 3.2. An EC‐Related Signature Was Developed From EndMT‐Associated Coexpression Networks and EC Gene Signatures in HCC

To screen the coexpression module related to EndMT, we utilized the ssGSEA approach to assign scores to tumor samples from TCGA‐LIHC dataset for the EndMT gene set. Based on the median score, the samples were classified into high‐scoring and low‐scoring groups. WGCNA was then conducted on the top 50% of differentially expressed genes across all samples in TCGA‐LIHC datasets. First, we calculated and presented the scale‐free fitting index for different soft thresholds (Figure 2a). This was done to ensure that the constructed network adhered more closely to a scale‐free topology. The results indicated that when the fitting index was set at 0.9, the minimum soft threshold that satisfied the construction of a scale‐free network was 6, which was thus determined as the optimal soft threshold. Subsequently, a coexpression network was constructed using this optimal soft threshold. The top 50% of differentially expressed genes were clustered using a clustering tree and labeled with relevant grouping information. Genes with the top 50% variance were grouped into 14 distinct modules. Next, the top 50% of variance genes were clustered, and the relationship between these genes and the merged modules was visualized (Figure 2b). Finally, based on the expression patterns of module genes, the correlations between all genes within the 14 modules and the EndMT high‐scoring and low‐scoring groups were determined (Figure 2c). Modules with a significant *p* value (< 0.05) were selected as criteria. Genes within one such module, specifically the MEblue module, were then screened for subsequent analysis. A total of 526 module genes were obtained from this module (Table S4). Furthermore, we screened for marker genes of malignant EC in PT, PVTT, and MLN according to the criteria of “*p*_val_adj < 0.05 and avg_log2FC > 1” (Table S4). Subsequently, univariate analysis was performed in combination with the survival information from TCGA‐LIHC dataset, and Cox genes with *p* < 0.05 were identified (Table S4). The intersecting genes among these three gene sets were defined as key gene signatures and finally obtained 19 genes (Figure 2d), which were screened based on EC and potentially affected the occurrence and development of HCC through the EndMT mechanism.

Figure 2Identification and validation of EC‐related prognostic genes in HCC. (a) Analysis of soft‐thresholding power to ensure scale‐free network topology in WGCNA. (b) Cluster dendrogram of gene modules identified by WGCNA, with different colors representing different modules. (c) Heatmap showing correlations between WGCNA modules and EC‐related traits. The blue module is significantly associated with EC characteristics. (d) Venn diagram illustrates the overlap among EC marker genes identified from scRNA‐seq, genes from the blue WGCNA module, and prognostic genes from TCGA‐LIHC datasets, resulting in the identification of 19 candidate genes. (e) LASSO regression trace plot showing coefficient profiles of the 19 candidate genes. (f) 10‐fold cross‐validation for tuning parameter (lambda) selection in LASSO regression. (g) Bar plot of LASSO‐derived coefficients for the nine‐gene EC‐related prognostic model. (h–j) Kaplan–Meier survival analysis for each of the nine genes in the prognostic model across three independent HCC cohorts: (h) TCGA‐LIHC, (i) GSE14520, and (j) ICGC‐LIRI‐JP.

**Figure:**
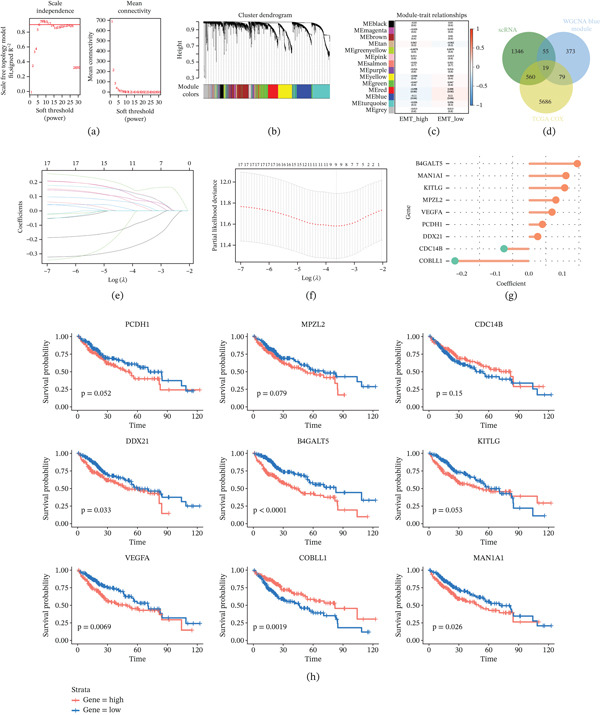
(A)

**Figure:**
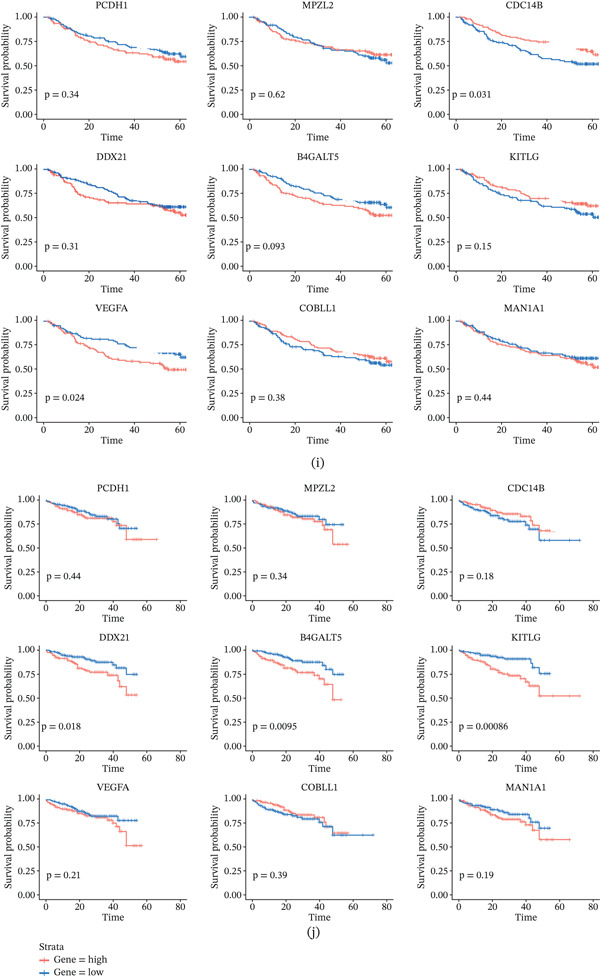
(B)

We included 19 genes in the LASSO regression analysis. To further evaluate the prognostic implications of these genes in TCGA‐LIHC, we conducted LASSO regression analysis and established a LASSO regression model. LASSO variable trajectory diagram (Figure 2e) and LASSO regression model diagram (Figure 2f) were drawn for visualization. The findings showed that the LASSO regression model comprised nine genes: PCDH1, MPZL2, CDC14B, B4GALT5, KITLG, VEGFA, COBLL1, DDX21, and MAN1A1. The EC‐related risk score (ECRS) of a sample could be generated using the expression and coefficients of these nine ECRGs, as shown in Figure 2g. Subsequently, we plotted the Kaplan–Meier curves for these nine genes in TCGA‐LIHC and validation cohorts (Figure 2h–j). In addition, ECRG scores were quantified at the single‐cell level, and cells were stratified into low‐ and high‐ECRG score subgroups. We then compared the cellular composition between the two groups and conducted GO and KEGG functional enrichment analyses based on the DEGs between the subgroups (Figure S1).

### 3.3. ECRS’s Independent Prognostic Role Was Validated in Multiple Cohorts

To evaluate the prognostic significance of the EC‐related signature, we calculated the ECRS for each sample in TCGA‐LIHC cohort and performed survival analyses by stratifying patients based on the median ECRS value. As shown in Figure 3a, patients in the low‐ECRS group exhibited significantly longer overall survival. This favorable prognosis in low‐ECRS individuals was further validated in multiple independent cohorts, including GSE14520 and ICGC‐LIRI‐JP. Time‐dependent ROC curves were generated to assess the predictive power of ECRS, and the area under the curve (AUC) at various time points indicated robust prognostic accuracy across both TCGA‐LIHC and external validation datasets (Figure 3b). To determine whether ECRS could serve as an independent prognostic factor for LIHC, univariate and multivariate Cox regression analyses were conducted. Results demonstrated that the prognostic impact of ECRS remained significant regardless of other clinical variables, supporting its role as an independent predictor (Figure 3c). A prognostic nomogram was subsequently developed by integrating ECRS with other independent clinical factors (stage, Child–Pugh score, AFP, age, and gender) to quantitatively estimate patient survival, thereby enhancing the clinical utility of ECRS (Figure 3d). The calibration curves showed strong agreement between predicted and observed survival probabilities at 1, 3, and 5 years, indicating reliable predictive performance (Figure 3e). Although ECRS was strongly associated with patient prognosis, its relationship with clinicopathologic features warranted further investigation. Histogram analyses revealed that patients with high ECRS scores were more likely to present with elevated AFP levels, macrovascular invasion, and advanced tumor stages (Figure 3f–h). Collectively, these findings suggest that ECRS not only serves as a reliable prognostic indicator for LIHC but is also significantly correlated with key clinicopathological parameters, underscoring its potential in clinical risk stratification.

Figure 3Prognostic performance and clinical relevance of the EC‐related gene signature in HCC. (a) Kaplan–Meier survival analysis of the EC‐related risk model across three independent cohorts (TCGA‐LIHC, GSE14520, and ICGC‐LIRI‐JP). Patients were stratified into high‐ and low‐risk groups based on the risk score. (b) Time‐dependent ROC curves demonstrating the predictive performance of the risk model at 1, 3, and 5 years in TCGA‐LIHC, GSE14520, and ICGC‐LIRI‐JP cohorts. (c) The forest plot shows multivariate Cox regression analyses evaluating the independent prognostic value of the ECRS along with clinical variables (age, gender, AFP, CTP, and stage). (d) Prognostic nomogram incorporating the EC‐related risk score and clinical features to predict 1‐, 3‐, and 5‐year overall survival in HCC patients. (e) Calibration curves of the nomogram at 1, 3, and 5 years. (f–h) Correlations between the EC‐related risk groups and clinical/pathological features: (f) vascular invasion (micro vs. macro), (g) tumor stage (I/II vs. III/IV), and (h) AFP level (< 200 vs. ≥ 200 ng/mL).

**Figure:**
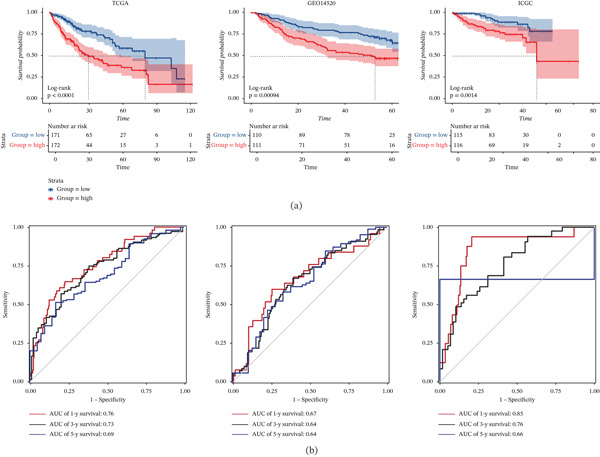
(A)

**Figure:**
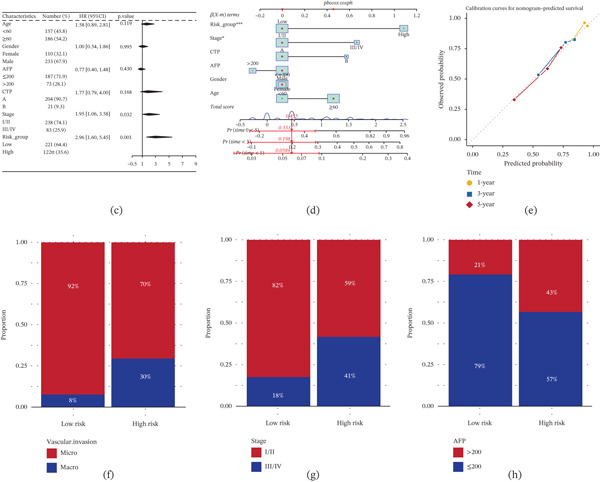
(B)

### 3.4. Construction of ECRGs Associated With Prognostic Subtypes

**Figure 4:**
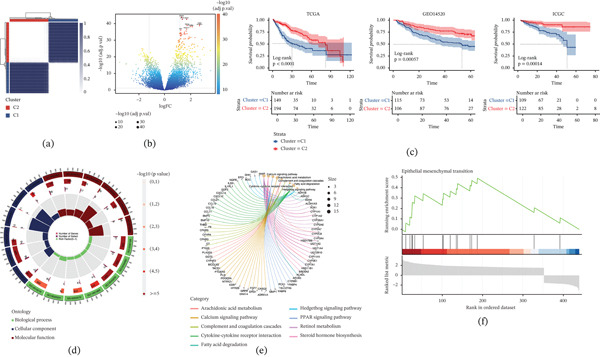
(a) Plot of consensus clustering results for LIHC samples from TCGA‐LIHC. (b) A volcano plot shows the DEGs of two subtypes from TCGA‐LIHC. (c) Kaplan–Meier curves comparing the two subtypes within TCGA‐LIHC, GSE14520, and ICGC‐LIRI‐JP cohorts. (d–f) GO, KEGG, and GSEA enrichment analyses of DEGs between two subtypes in from TCGA‐LIHC.

To investigate potential molecular subtypes within the LIHC samples, ConsensusClusterPlus was employed to perform consensus clustering based on the expression profiles of ECRGs. This analysis identified two distinct subtypes of LIHC (Figure 4a), designated as Subtype A (Cluster 1) and Subtype B (Cluster 2). A volcano plot was used to visualize the differentially expressed genes between the two subtypes in TCGA‐LIHC (Figure 4b). KM survival analysis revealed a significant difference in overall survival between the two groups in TCGA‐LIHC and the validation cohorts, with Subtype B showing a more favorable prognosis (*p* < 0.05, Figure 4c).

The underlying reasons and mechanisms by which ECRS significantly influences LIHC prognosis remain to be fully elucidated. To explore this, GO, KEGG, and GSEA analyses were performed on the DEGs between LIHC subtypes in TCGA‐LIHC cohort. The results revealed that these DEGs were involved in multiple metastasis‐related pathways, including epithelial–mesenchymal transition (EMT), PPAR signaling, calcium signaling, cytokine–cytokine receptor interaction, and Hedgehog signaling (Figure 4d–f).

### 3.5. Significant Differences in EndMT Between ECRG‐Based Prognostic Subtypes

To investigate the relationship between EndMT and ECRG‐based prognostic subtypes, we analyzed the expression patterns of EndMT‐related genes across three independent cohorts: TCGA‐LIHC, GSE14520, and ICGC‐LIRI‐JP. Heatmaps (Figure S2a–c) demonstrated clear differences in the expression of EndMT‐related genes between the subtypes, with distinct clustering observed in each dataset. Notably, genes such as MMP2, COL1A1, and ACTA2 showed higher expression in the high‐risk subtypes, indicating elevated EndMT activity. We next performed correlation analyses among EndMT genes within each dataset (Figure S2d–f). The results showed strong positive correlations between canonical EndMT markers, particularly among MMP9, FN1, and COL3A1, suggesting coordinated regulation in high‐EndMT subtypes. This pattern was consistently observed across all three datasets, reinforcing the robustness of the prognostic subtypes in prognostic stratification. In addition, we assessed the association between ECRG‐based subtypes and hallmark pathways (Figure S2g–i). The high‐risk subtypes exhibited stronger positive correlations with hallmark pathways such as EMT, angiogenesis, and TGF‐*β* signaling, further supporting the involvement of EndMT‐related biological processes in the poor prognosis group. Together, these results suggest that ECRG‐based subtypes are closely related to EndMT and can effectively predict the prognosis of patients with HCC.

### 3.6. Differences in TIDE Scores, Immune Checkpoints, and Immune Infiltration Between ECRG‐Based Prognostic Subtypes

**Figure 5:**
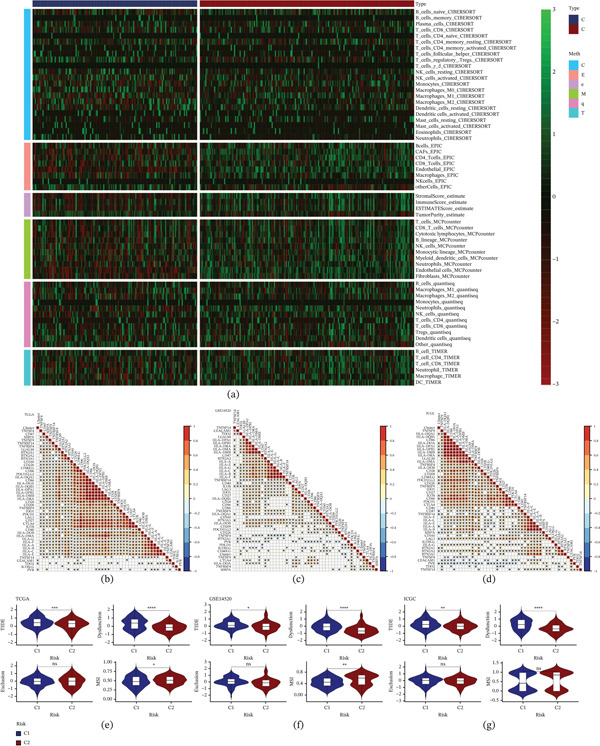
(a) Heatmap showing immune infiltration patterns across different subtypes as estimated by various algorithms in TCGA‐LIHC cohort. (b–d) Correlation heatmaps illustrating the relationships between immune checkpoint genes and different subtypes across various LIHC datasets. (e–g) TIME scores for different subtypes across three independent datasets.

To elucidate the immune landscape of ECRG‐based prognostic subtypes, we conducted a comprehensive immune infiltration analysis in TCGA‐LIHC cohort. As shown in Figure 5a, there were differences in the immune cell infiltration characteristics between the C1 and C2 subtypes. The C2 subtype exhibited higher levels of various immune cells, including CD8+ T cells, macrophages, and dendritic cells, across multiple computational algorithms (CIBERSORT, EPIC, MCPcounter, TIMER, etc.), suggesting a more immune‐active TME. Correlation analysis of immune checkpoint gene expression revealed substantial differences between subtypes across TCGA‐LIHC, GSE14520, and ICGC‐LIRI‐JP datasets (Figure 5b–d). The C2 subtype showed stronger positive correlations among key immune checkpoint molecules, such as PDCD1 (PD‐1), CTLA4, LAG3, and TIGIT, implying potential immune evasion mechanisms in high‐risk patients. To further assess the functional relevance of immune differences, we compared TIDE scores between subtypes (Figure 5e–g). Across all datasets, the C2 subtype displayed significantly higher TIDE, exclusion, and dysfunction scores, indicating a higher likelihood of immune escape and potentially poorer response to immunotherapy. Collectively, these results demonstrate that ECRG‐based prognostic subtypes are associated with distinct immune landscapes, characterized by differences in immune infiltration levels, immune checkpoint gene interactions, and immune evasion potential, which may have implications for immunotherapeutic strategies in liver cancer.

### 3.7. Predicting Therapy Response and Drug Candidates Based on ECRGs

**Figure 6:**
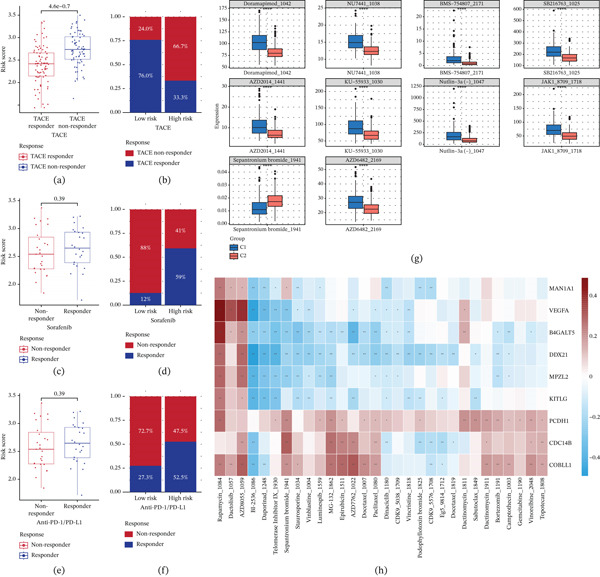
(a–f) Scatter plots and box plots illustrating ECRS in response to different therapies, as well as the proportions of treatment responses across various therapies between high and low ECRS groups. (g) IC50 values of the Top 10 most significantly different drugs in different subtypes from TCGA‐LIHC. (h) Heatmap showing the correlations between ECRGs and various drugs.

To evaluate the predictive value of ECRGs in therapeutic response, we analyzed patient responses to various treatments based on ECRG expression. As shown in Figure 6a–f, higher ECRG‐based risk scores were significantly associated with poorer response to TACE, sorafenib, and anti‐PD‐L1/PD‐1 therapy. Specifically, TACE responders showed significantly lower ECRG scores compared to nonresponders, and a greater proportion of high‐risk patients were TACE nonresponders. Similar trends were observed in sorafenib and anti‐PD‐L1/PD‐1‐treated cohorts, where nonresponders exhibited higher ECRG scores and high‐risk groups were more frequently nonresponsive. We further investigated potential therapeutic agents that may be differentially effective between the two ECRG‐based prognostic subtypes. Drug sensitivity analysis revealed several compounds with significantly different predicted responses between subtypes (Figure 6g). Notably, compounds such as AZD8055, doxorubicin, nutlin‐3a, and sepantronium bromide showed higher sensitivity in the C1 subtype, suggesting these may represent more effective therapeutic options for this group. Finally, correlation analysis between ECRG expression and sensitivity to a panel of anticancer drugs identified several key genes associated with drug response (Figure 6h). For instance, MAN1A1 and VEGFA were positively correlated with resistance to multiple agents, while genes like CDH1 and MPZL2 showed negative correlations with drug resistance, suggesting their potential roles in modulating treatment efficacy. These findings highlight the utility of ECRGs in predicting response to conventional and targeted therapies and offer insight into potential personalized treatment strategies for different molecular subtypes of liver cancer.

### 3.8. HCC Organoid Model Reveals the Role of ECRGs in EndMT

**Figure 7:**
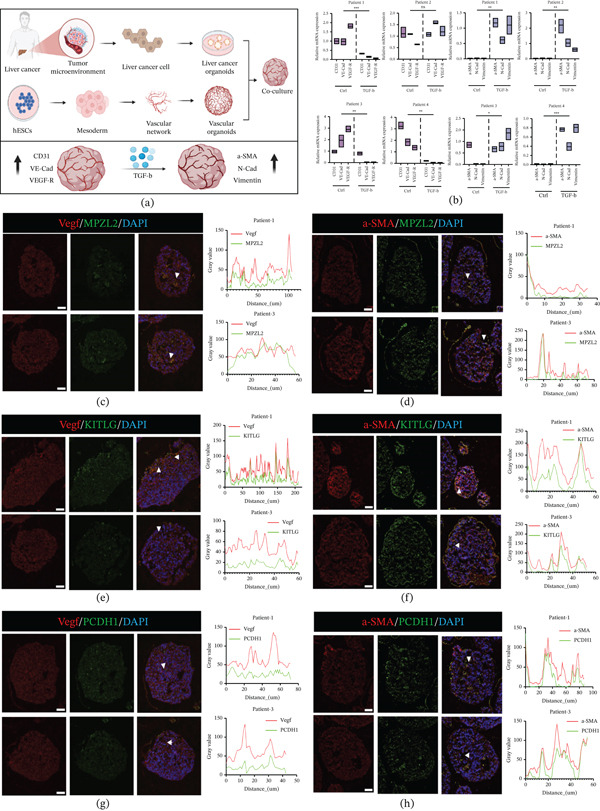
(a) Schematic diagram illustrating the construction of a vascularized HCC organoid model for studying EndMT. (b) RT‐qPCR analysis showing changes in endothelial markers (CD31, VE‐cadherin, and VEGFR) and mesenchymal markers (*α*‐SMA, N‐cadherin, and vimentin) after TGF‐*β* treatment in organoids derived from four patients. (c–h) Immunofluorescence staining demonstrating the expression changes of endothelial markers (c) VEGFR and MPZL2, (e) KITLG, and (g) PCDH1, as well as mesenchymal markers (d) *α*‐SMA and MPZL2, (f) KITLG, and (h) PCDH1 in cocultured liver cancer organoids. Scale bars represent 50 *μ*m. Colocalization was quantified using ImageJ. ns: *p* > 0.05,  ^∗∗^ *p* < 0.01,  ^∗∗∗^ *p* < 0.001.

As shown in Figure 7a, a coculture system comprising liver cancer organoids and vascular organoids was successfully established using tumor tissues from four patients with HCC. Briefly, liver cancer tissues were obtained from HCC patients, and tumor cells were isolated and cultured to generate liver cancer organoids. In parallel, hESCs were induced to differentiate into the mesodermal lineage to form vascular networks and vascular organoids. The two organoid types were subsequently cocultured to create a model that closely mimics the TME of HCC. To induce EndMT, TGF‐*β* treatment was applied. RT‐qPCR analysis revealed a significant downregulation of endothelial markers CD31, VE‐cadherin, and VEGF‐R, alongside a marked upregulation of mesenchymal markers *α*‐SMA, N‐cadherin, and vimentin (Figure 7b), confirming successful induction of EndMT and providing a foundation for subsequent mechanistic studies. Immunofluorescence analysis was conducted to investigate the spatial relationship between ECRGs and EndMT markers in samples from Patient 1 and Patient 3. Notably, MPZL2, KITLG, and PCDH1 were found to colocalize with both mesenchymal marker *α*‐SMA and endothelial marker VEGF (Figure 7c–h), suggesting potential roles for these genes in EndMT and modulation of the TME.

## 4. Discussion

HCC poses a significant global health challenge due to its high mortality and increasing incidence rates. Understanding the mechanisms underlying HCC progression is essential for developing effective therapeutic strategies. Our study elucidates the critical role of ECs in HCC progression, highlighting their transformation into mesenchymal cells through EndMT. This transformation not only contributes to tumor metastasis but also correlates with poor patient prognosis [38]. Importantly, we identified a unique endothelial cell–related gene signature that serves as an independent prognostic indicator in HCC, validated across multiple cohorts. Our findings emphasize the clinical relevance of ECs in HCC, demonstrating that the ECRS can effectively stratify patients based on survival outcomes. Moreover, our comprehensive bioinformatics analyses provide a robust methodological framework, and the use of organoid models further validated the strong association between specific ECRGs and EndMT, highlighting the innovative nature of our study. These insights into the EndMT mechanism and its prognostic implications may pave the way for targeted therapeutic strategies, thereby improving the clinical management of HCC.

The study identified nine key genes associated with ECs that play significant roles in the progression of HCC. Among these, PCDH1 is known for its involvement in cell adhesion and signaling, which may influence TME interactions and metastasis [39]. MPZL2 is implicated in cell adhesion and may contribute to the invasive properties of breast cancer cells [40]. CDC14B is involved in cell cycle regulation, suggesting its potential role in tumor proliferation [41]. B4GALT5 is associated with glycosylation processes that can affect cell signaling and tumor progression [42]. KITLG is a growth factor that may promote angiogenesis and tumor growth, highlighting its relevance in HCC ([43]). VEGFA is a well‐known angiogenic factor that facilitates blood vessel formation, crucial for tumor survival and growth [44]. COBLL1 is involved in cytoskeletal organization, which may affect cell motility and invasion [45]. MAN1A1 is linked to glycoprotein processing, potentially influencing tumor cell behavior [46]. The LASSO regression analysis revealed that these genes collectively form a prognostic model, indicating that their expression levels correlate with patient survival outcomes. The identification of these genes underscores the importance of ECs in HCC progression and suggests that targeting these pathways may provide therapeutic opportunities. The findings align with existing literature that emphasizes the role of endothelial dysfunction and TME in cancer progression, reinforcing the potential of these genes as biomarkers for prognosis and targets for intervention in HCC treatment.

The results of this study highlight the significant involvement of ECRGs in various signaling pathways, particularly EMT and cytokine–cytokine receptor interactions, which are crucial in the progression and metastasis of HCC. EMT is a biological process that enables epithelial cells to acquire mesenchymal properties, facilitating tumor invasion and metastasis [47]. The upregulation of mesenchymal markers in ECs, as observed in our analysis, suggests that these cells may contribute to the metastatic potential of HCC through the EndMT mechanism. Additionally, the cytokine–cytokine receptor interaction pathway plays a pivotal role in mediating the communication between tumor cells and the surrounding microenvironment, influencing tumor growth and immune evasion [48, 49]. The findings indicate that the EC‐related signature developed in this study can serve as a prognostic tool, reflecting the aggressive nature of HCC and its propensity for metastasis. By elucidating the pathways involved in HCC progression, this research underscores the potential for targeting these pathways therapeutically, offering new avenues for intervention in HCC management. The identification of key genes associated with these pathways not only enhances our understanding of HCC biology but also provides a foundation for developing novel therapeutic strategies aimed at improving patient outcomes in this challenging malignancy.

The immune analysis results indicate significant differences in immune cell infiltration, immune checkpoint gene expression, and immune evasion mechanisms associated with EC‐related prognostic subtypes in HCC. Specifically, the presence of tumor‐infiltrating ECs correlates with a favorable prognosis and suggests a role in the progression of HCC through EndMT. The study’s findings highlight that high levels of EC infiltration are linked to enhanced immune activity, characterized by increased infiltration of CD8+ T cells and other immune cells, which may contribute to a more effective antitumor response [50]. Furthermore, the differential expression of immune checkpoint genes between EC‐related subtypes underscores the potential for tailored immunotherapeutic strategies in HCC treatment. The identification of a nine‐gene signature associated with ECs provides a novel prognostic tool that could aid in risk stratification and therapeutic decision‐making for HCC patients. Importantly, the EC‐related signature was validated in a cohort of patients receiving immunotherapy, further supporting its clinical relevance. In addition, EC‐related subtypes were also found to stratify therapeutic responses to sorafenib and TACE, emphasizing the critical role of EC‐associated signatures in shaping immune responses and influencing tumor progression in HCC.

The coculture system of liver cancer and vascular organoids provides a physiologically relevant model that recapitulates the complex interactions between tumor and endothelial cells within the TME. This 3D platform enables dynamic investigation of EndMT processes under tumor‐derived stimuli, offering novel insights into tumor progression, vascular remodeling, and potential therapeutic interventions targeting EC plasticity in HCC. Our findings reveal that three ECRGs—MPZL2, KITLG, and PCDH1—show spatial colocalization with EndMT‐related markers, suggesting their potential roles as key targets for modulating EndMT and intervening in tumor‐associated vascular transformation.

This study has several limitations that warrant consideration. First, the relatively small sample size may limit the generalizability of our research results. Meanwhile, the differences in RNA/protein levels of key genes have not been verified in different tissues. Therefore, large‐scale clinical validation is needed to confirm the role of EC‐related genes in HCC and their potential clinical applications. Furthermore, the use of multiple datasets may introduce batch effects, complicating the interpretation of results across different cohorts. Despite these limitations, our findings indicate that tumor‐infiltrating ECs are associated with prognosis and play a significant role in HCC progression through EndMT. The development of an ECRS demonstrates its potential as an independent prognostic factor, correlating with key clinicopathological features. Future research should focus on validating these results in larger cohorts and exploring the therapeutic implications of ECRS in personalized treatment strategies for HCC patients.

## Author Contributions

Z.C. and Y.C. contributed to the study concept and design. F.W. and X.Y. wrote the first draft of the manuscript. X.Y. and Zhi.C. contributed to the statistical analysis. All authors made substantial contributions to conception and design, acquisition of data, or analysis and interpretation of data; and took part in drafting the article or revising it critically for important intellectual content. F.W. and X.Y. contributed equally to this study.

## Funding

The study was funded by Startup Fund for Scientific Research of Fujian Medical University (2023QH1094), Joint Funds for the Innovation of Science and Technology, Fujian Province (2024Y93011223), and University‐Industry Research Cooperation Project of Science and Technology, Fujian Province (2024Y41010089).

## Disclosure

All authors agreed to submit to the current journal, gave final approval of the version to be published, and agreed to be accountable for all aspects of the work.

## Ethics Statement

This study was approved by the Ethics Committee of the First Affiliated Hospital of Fujian Medical University (MRCTA, ECFAH of FMU [2024] 469), and the requirement for individual informed consent was waived due to the retrospective nature of the analysis and the use of deidentified data.

## Consent

All authors have agreed to publish this manuscript.

## Conflicts of Interest

The authors declare no conflicts of interest.

## Supporting Information

Additional supporting information can be found online in the Supporting Information section.

## Supporting information


**Supporting Information 1** Table S1: List of immune checkpoint genes.

**Supporting Information 2** Table S2: Primer sequences used in RT‐qPCR analysis.

**Supporting Information 3** Table S3: List of antibodies used for immunofluorescence staining and their catalog numbers.

**Supporting Information 4** Table S4: List of genes associated with prognosis in LIHC and ECs.

**Supporting Information 5** Figure S1: Analysis of ECRG signature in single‐cell RNA sequencing data. (a) Distribution of major cell types across the low‐ and high‐ECRG score groups. Cell proportions were visualized using stacked bar plots. The ECRG score for each cell was calculated based on the ECRG signature using the AUC algorithm implemented in the AUCell package, and cells were stratified into low‐ and high‐score groups according to the median ECRG score. (b) GO and (c) KEGG enrichment analysis of DEGs between the two ECRG score groups. Figure S2: (a–c) Heatmaps showing the expression levels of immune checkpoint genes across different subtypes in three independent datasets. (d–f) Correlation analyses of immune checkpoint gene expression in the corresponding subtypes. (g–i) Correlation analyses between two subtypes and hallmark pathways across the three independent datasets.

## Data Availability

The datasets analyzed for this study can be found in TCGA database (http://www. http://cancer.gov/tcga), the Gene Expression Omnibus (https://www. ncbi. http://nlm.nih.gov/geo), and the International Cancer Genome Consortium (https://dcc.icgc.org/releases/current/Projects).
