# Supplementary material for: Integrated Single‐Cell and Bulk Transcriptomic Analysis Reveals an Endothelial Gene Signature Shaping EndMT and Prognosis in Hepatocellular Carcinoma
Source: Hum Mutat. 2026 Jun 17;2026:4041113. doi: 10.1155/humu/4041113 (PMC13275994; doi:10.1155/humu/4041113)
Supplement: Supplementary file 5 — Supporting Information 5 Figure S1: Analysis of ECRG signature in single‐cell RNA sequencing data. (a) Distribution of major cell types across the low‐ and high‐ECRG score groups. Cell proportions were visualized using stacked bar plots. The ECRG score for each cell was calculated based on the ECRG signature using the AUC algorithm implemented in the AUCell package, and cells were stratified into low‐ and high‐score groups according to the median ECRG score. (b) GO and (c) KEGG enrichment analysis of DEGs between the two ECRG score groups. Figure S2: (a–c) Heatmaps showing the expression levels of immune checkpoint genes across different subtypes in three independent datasets. (d–f) Correlation analyses of immune checkpoint gene expression in the corresponding subtypes. (g–i) Correlation analyses between two subtypes and hallmark pathways across the three independent datasets. [file HUMU-2026-4041113-s004.docx]

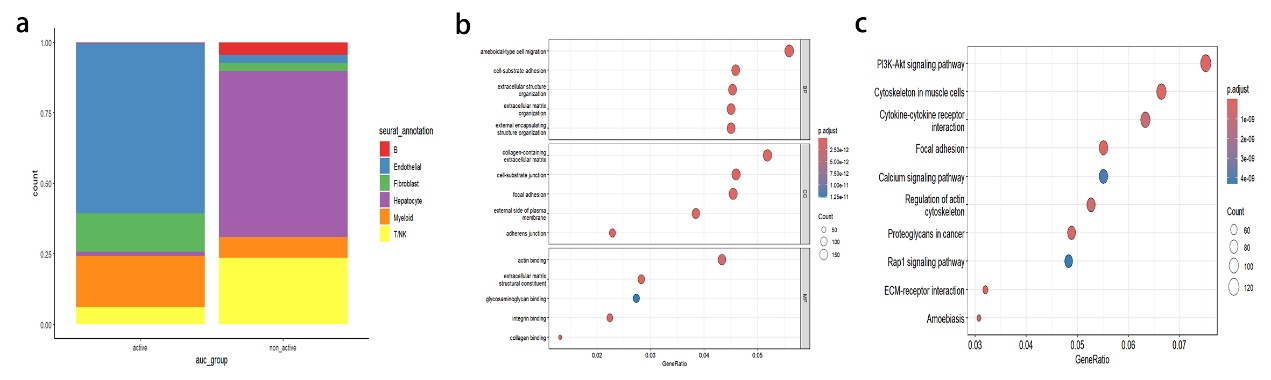


**Figure S1.** Analysis of ECRG Signature in single-cell RNA sequencing data. (a) Distribution of major cell types across the low- and high-ECRG score groups. Cell proportions were visualized using stacked bar plots. The ECRG score for each cell was calculated based on the ECRG signature using the AUC algorithm implemented in the AUCell package, and cells were stratified into low- and high-score groups according to the median ECRG score. GO(b) and KEGG(c) enrichment analysis of DEGs between the two ECRG score groups.


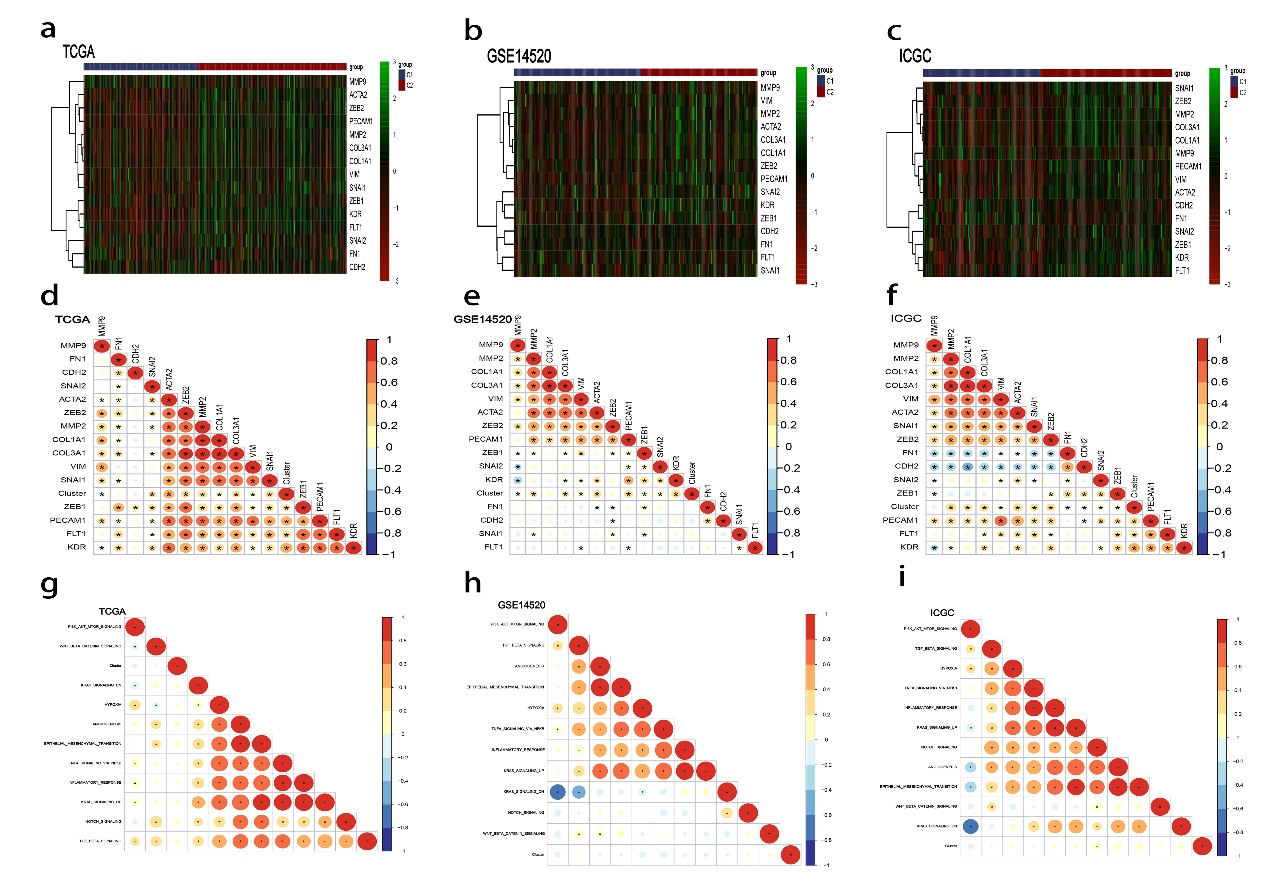


**Figure S2.** (a-c) Heatmaps showing the expression levels of immune checkpoint genes across different subtypes in three independent datasets. (d-f) Correlation analyses of immune checkpoint gene expression in the corresponding subtypes. (g-i) Correlation analyses between two subtypes and hallmark pathways across the three independent datasets.
